# Si_3_N_4_ Nanoparticle Reinforced Si_3_N_4_ Nanofiber Aerogel for Thermal Insulation and Electromagnetic Wave Transmission

**DOI:** 10.3390/gels11050324

**Published:** 2025-04-26

**Authors:** Zongwei Tong, Xiangjie Yan, Yun Liu, Yali Zhao, Kexun Li

**Affiliations:** 1 Department of Materials Science and Engineering, Jinzhong University, Jinzhong 030619, China; yxja899@163.com (X.Y.); yaliz12@163.com (Y.Z.); likexuncc@126.com (K.L.); 2 School of Material Science and Engineering, Tianjin University, Tianjin 300072, China; ly1995@tju.edu.cn

**Keywords:** silicon nitride, nanofiber aerogel, particle reinforcement, thermal insulation, electromagnetic wave transmission

## Abstract

Traditional nanoparticle aerogels suffer from inherent brittleness and thermal instability at elevated temperatures. In recent years, ceramic nanofiber aerogels, utilizing flexible nanofibers as structural units, have emerged as mechanically resilient alternatives with ultrahigh porosity (>90%). However, their thermal insulation capabilities are compromised by micron-scale pores (10–100 μm) and overdependence on ultralow density, which exacerbates mechanical fragility. This study pioneers a gas-phase self-assembly strategy to fabricate Si_3_N_4_ nanoparticle reinforced Si_3_N_4_ nanofiber aerogels (SNP-R-SNFA) with gradient pore architectures. By leveraging methyltrimethoxysilane/vinyltriethoxysilane composite aerogel (MVa) as a reactive template, we achieved spontaneous growth of Si_3_N_4_ nanofiber films (SNP-R-SNF) featuring nanoparticle-fiber interpenetration and porosity gradients. The microstructure formation mechanism of SNP-R-SNF was analyzed using field-emission scanning electron microscopy. Layer assembly and hot-pressing composite technology were employed to prepare the SNP-R-SNFA, which showed low density (0.033 g/cm^3^), exceptional compression resilience, insensitive frequency dependence of dielectric properties (ε′ = 2.31–2.39, tan δ < 0.08 across 8–18 GHz). Infrared imaging displayed backside 893 °C cooler than front, demonstrating superior insulation performance. This study not only provides material solutions for integrated electromagnetic wave-transparent/thermal insulation applications but more importantly establishes an innovative paradigm for enhancing the mechanical robustness of nanofiber-based aerogels.

## 1. Introduction

Traditional aerogels employ the chain-like assembly of nanoparticles to construct pearl-necklace-like architectures, which subsequently form three-dimensional highly porous networks through interconnected nanoparticle chains [1,2]. Although their thermal conductivity is comparable to that of air, their extremely low mechanical properties have long troubled researchers [3,4]. Ceramic aerogels (such as Si_3_N_4_ and SiC) are typically prepared through physicochemical transformations of precursor aerogels (e.g., C/SiO_2_ binary aerogels) under high-temperature conditions [5,6,7]. However, the high temperature inevitably triggers the pearl-necklace-like structure to be prone to sintering, which not only increases the skeleton size but also reduces porosity, significantly elevating the solid-phase thermal conductivity. Consequently, research reports on particulate Si_3_N_4_ and SiC aerogels have gradually declined.

In response to these challenges, ceramic nanofiber aerogels have emerged as mechanically resilient alternatives, where flexible ceramic nanofibers replace nanoparticles as fundamental building blocks [8,9]. These three-dimensional architectures demonstrate remarkable compressive resilience (>80% strain recovery) while maintaining porosity exceeding 90% [10,11]. However, some of the current research work unnecessarily includes a lot of flashy data, merely for showcasing richness, while lacking consideration of the material’s practical application significance. For example, imparting hydrophobicity to Si_3_N_4_ nanofiber aerogels through surface modification and testing their oil-water separation capability may yield satisfactory results [12,13], but in practice, many inexpensive adsorbent materials can perform this task. Using Si_3_N_4_ nanofiber aerogels in such applications is evidently unwise.

Si_3_N_4_ ceramic possesses high strength, corrosion resistance, oxidation resistance, and a moderate dielectric constant, making it material with significant potential for electromagnetic wave transmission [14,15]. Si_3_N_4_ nanofiber aerogels exhibit excellent intrinsic properties of Si_3_N_4_ while also demonstrating ultralow thermal conductivity, making them particularly suitable for hypersonic vehicle radomes requiring simultaneous microwave transparency and thermal insulation. Preparing nanoscale, high aspect ratio Si_3_N_4_ nanofibers is the key to assembling elastic Si_3_N_4_ nanofiber aerogels. Chemical vapor deposition technology is widely used to prepare Si_3_N_4_ nanofibers, followed by layer-by-layer self-assembly or freeze-forming to obtain Si_3_N_4_ nanofiber aerogels [16,17]. Nevertheless, unlike particulate aerogels with nanoscale pores (20–50 nm), the pore diameter of nanofiber aerogels typically reaches several micrometers or even tens of micrometers, rendering them susceptible to convective heat transfer enhancement under turbulent airflow conditions, which will greatly affect their thermal insulation performance. Our previous work addressed this limitation through in situ growth of SiO_x_ nanowires on Si_3_N_4_ nanofibers surfaces via low-temperature chemical vapor deposition technology, effectively subdividing micrometer-sized pores into submicron domains [18]. However, after the operating temperature exceeds 1000 °C, the SiO_x_ nanowires will burn out, leading to increased pore size again. Moreover, due to the soft characteristics of nanofiber aerogels, incorporating other thermal insulation materials within their pores is also very challenging, and few related research reports are available.

In another of our studies on organosilane-derived Si_3_N_4_ aerogels, we prepared Si_3_N_4_ aerogels by treatment of organosilane aerogels at 1450 °C and accidentally discovered some Si_3_N_4_ nanofiber film on the surface of the Si_3_N_4_ aerogel. The characterization results showed that the two sides of Si_3_N_4_ nanofiber film possess different microstructures and compositions. The side near the sample is composed of Si_3_N_4_ nanofibers and Si_3_N_4_ nanoparticles, while the outer side of the film consists only of Si_3_N_4_ nanofibers. That is very interesting. Inspired by this observation, the present study employed thermal lamination of Si_3_N_4_ nanofiber films through layer-by-layer assembly, ultimately fabricating a hierarchical Si_3_N_4_ nanofiber aerogel architecture. This material, designated as Si_3_N_4_ nanoparticles reinforced Si_3_N_4_ nanofiber aerogel (SNP-R-SNFA), demonstrates low density, exceptional compressive resilience, low dielectric constant and dielectric loss, and superior thermal insulation performance. It is expected to serve as a flexible thermal insulation liner for high-temperature resistant electromagnetic wave-transparent antenna covers, significantly enhancing thermal insulation performance while ensuring normal transmission of electromagnetic wave signals through the radome of high Mach-number aircraft. This paper provides theoretical support and technical basis for promoting the development of novel ceramic nanofiber aerogels, possessing an important scientific research value.

## 2. Results and Discussion

### 2.1. Microstructure Formation Mechanism

**Figure 1:**
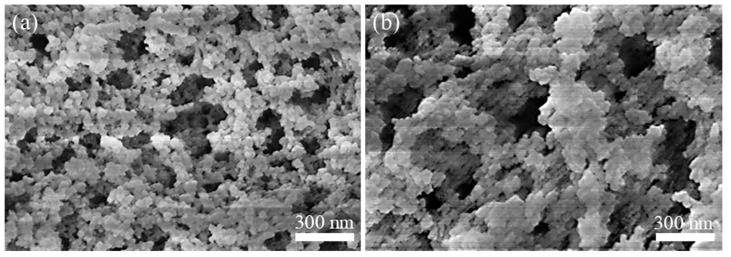
SEM images of (**a**) MTa and (**b**) MVa.

In this study, two types of organosilane aerogels were initially synthesized as parent matrices for generating Si_3_N_4_ nanofiber films, aiming to compare the effects of the microstructure and composition of organosilane aerogels on the microstructure of Si_3_N_4_ nanofiber films: a pure methyltrimethoxysilane (MTMS) aerogel and a composite aerogel with a molar ratio of MTMS to vinyltriethoxysilane (VTES) of 2:1, which were named MTa and MVa, respectively. Results revealed that, under identical processing conditions, the Si_3_N_4_ nanofiber film generated on the surface of MVa exhibited greater thickness and easier peelability from the parent aerogel relative to that of MTa. As shown in the SEM images of MTa and MVa (Figure 1a,b), MTa displayed a homogeneous network of nanoscale pores, whereas MVa, subjected to steric hindrance effects from vinyl functional groups during the sol-gel transition [19], developed a bimodal pore-size distribution.

Consequently, during high-temperature reaction processes, MTa experienced accelerated densification through sintering, which induced pore collapse and the consequent eradication of sustained gas retention zones (SiO, CO, and N_2_) at its surface. This resulted in localized and discontinuous Si_3_N_4_ nanofibers growth on MTa, thereby precluding the development of a continuous Si_3_N_4_ nanofiber film. In contrast, based on our previous research [20], the conversion of MVa into Si_3_N_4_ aerogel followed a vapor-phase trimming-induced microstructural refinement mechanism. By comparing Figure 1a,b, it can be observed that MVa exhibits a larger pore size, which contributes to its enhanced resistance to instantaneous sintering under identical thermal treatment conditions. Progressive pore contraction, synergistically coupled with three-dimensional skeletal restructuring, dynamically regenerated interconnected mesopores, thereby providing ample reaction space for Si_3_N_4_ nanofibers nucleation. The sustained gas retention zones established both internally and at the surface allowed continuous diffusion of reactive gases (SiO and CO) to the near-surface region, where they reacted with high-concentration N_2_ to accelerate the growth of Si_3_N_4_ nanofibers, ultimately forming a continuous Si_3_N_4_ nanofiber film.

**Figure 2:**
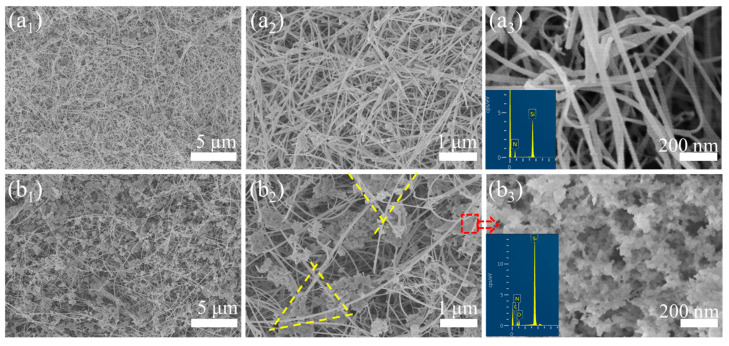
SEM images of P-side (**a_1_**–**a_3_**) and H-side (**b_1_**–**b_3_**) of Si_3_N_4_ nanofiber film.

During the delamination of the Si_3_N_4_ nanofiber membrane from the parent aerogel, the nanoparticles embedded within the Si_3_N_4_ aerogel matrix and interwoven with Si_3_N_4_ nanofibers were co-exfoliated, forming an Si_3_N_4_ nanoparticles-reinforced Si_3_N_4_ nanofibers (SNP-R-SNF) architecture. Intriguingly, the film displayed heterogeneous microstructural features across its dual surfaces: the parent aerogel-contacting interface was designated as the H (Hybrid) side characterized by nanoparticle-fiber interpenetration, while the opposing surface exhibited a P (Pure) side dominated by aligned nanofibers with minimal particulate incorporation. As evidenced by the SEM images of the P and H sides (Figure 2a_1_,b_1_) of the Si_3_N_4_ nanofiber membrane, the P-side was composed exclusively of densely interwoven Si_3_N_4_ nanofibers, while the H-side displayed a pearl-necklace-like architecture, in which Si_3_N_4_ nanofibers were intertwined with Si_3_N_4_ nanoparticles.

The higher density of SNP-R-SNF on the P-side compared to the H-side was attributed to its exposure to an unobstructed free space with elevated N_2_ concentration, which preferentially facilitated the growth of Si_3_N_4_ nanofibers. This theoretically supported the existence of a gradient distribution in nanofiber density across the Si_3_N_4_ nanofiber film, transitioning from the P-side to the H-side. In addition, from the high-magnification SEM images in Figure 2a_2_,b_2_, it can be found that the pores formed by the lap of Si_3_N_4_ nanofibers on the P-side were about 200 nm, while the H-side exhibited sparse Si_3_N_4_ nanofibers with near-micron-scale pores. Nonetheless, the nanoparticle aerogel in the pore further segmented most of the micron level pores. Crucially, nanoparticle-filled aerogels within these larger pores (as shown in Figure 2b_3_) subdivided them into sub-200 nm domains, mitigating adverse effects of micron-scale porosity. Based on this, MVa was employed as the parent aerogel for the preparation of SNP-R-SNF films in the subsequent studies.

**Figure 3:**
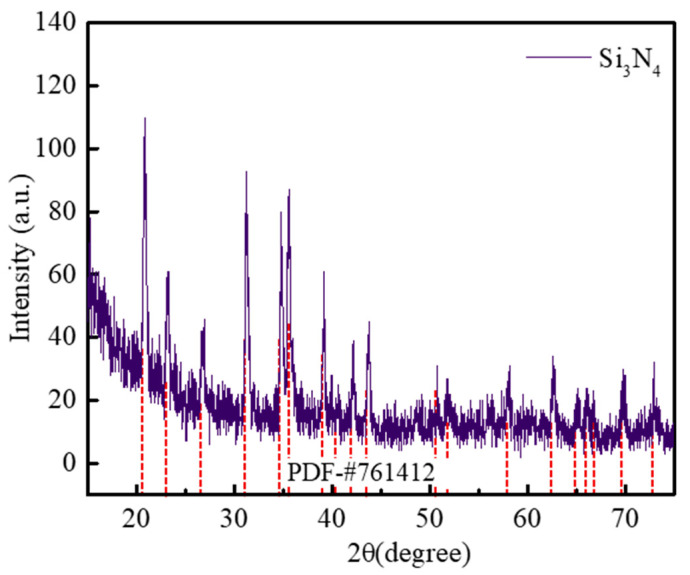
XRD pattern of SNP-R-SNFA.

As shown in Figure 3, the XRD pattern of the SNP-R-SNF film featured sharp diffraction peaks indexed to the α-Si_3_N_4_ crystalline phase (JCPDS No. 76-1412), alongside a weakly resolved peak centered at 20.1° corresponding to amorphous SiO_2_. This aligns with the EDS mapping in Figure 2b_3_, confirming incomplete carbothermal reduction in the organosilane aerogel, which left trace SiO_2_ and carbon (C) impurities. While the residual SiO_2_ phase contributes beneficially to impedance matching through its moderate permittivity, the graphitic carbon residues introduce undesirable dielectric losses that degrade electromagnetic wave transparency [21,22]. To mitigate this, the exfoliated SNP-R-SNF films were subjected to isothermal treatment at 900 °C for 3 h in dry air, effectively removing C contaminants.

**Figure 4:**
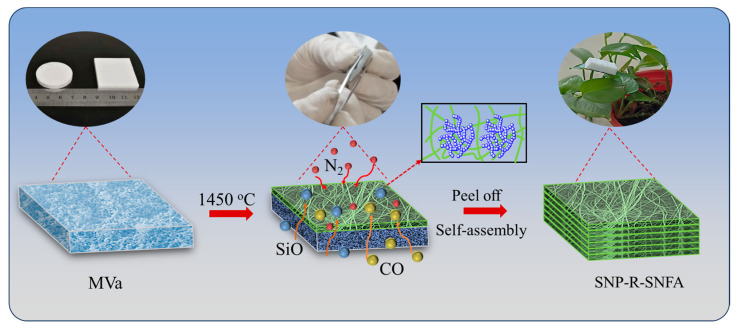
Schematic diagram of process route and formation mechanism of SNP-R-SNFA.

On the basis of the above investigations, the SNP-R-SNFA was fabricated via layer-by-layer assembly followed by hot-pressing of the composite SNP-R-SNF, with the detailed preparation process illustrated in Figure 4. It is critical to emphasize that the H-side must be oriented upward during layer assembly. When employed as a thermal insulation and electromagnetic wave transmission material, this upward-facing H-side will directly interact with electromagnetic waves and the thermal environment, leveraging its hybrid microstructure for enhanced performance.

### 2.2. Compression Performance and Reinforcement Mechanism

**Figure 5:**
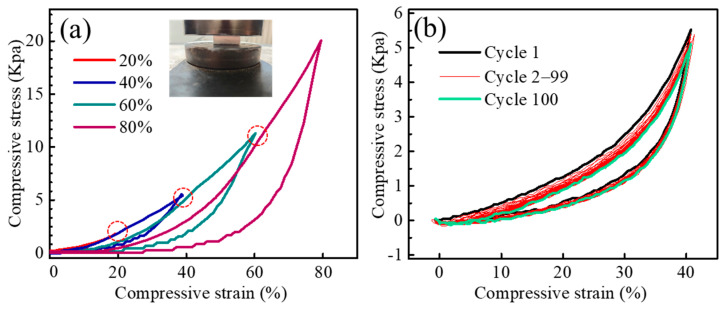
(**a**) Compressive stress-strain relationship curves under predefined strain conditions (20%, 40%, 60%, and 80%); (**b**) fatigue testing over 100 cycles under 40% compressive deformation.

To comprehensively investigate the compression resistance characteristics and underlying reinforcement mechanisms of SNP-R-SNFA, cyclic loading-unloading tests were conducted at four strain levels (20%, 40%, 60%, and 80%), as shown in Figure 5a. The corresponding maximum compressive strengths were measured as 1.8 kPa, 5.4 kPa, 11.3 kPa, and 20.1 kPa, respectively, which were higher than those of the pure Si_3_N_4_ nanobelt aerogel [23], Si_3_N_4_@SiOx nanofibrous aerogel [18], Si_3_N_4_@SiO_2_ nanofibre sponges [24], and so on. Building upon these findings, subsequent 100-cycle testing at 40% strain was performed, as visualized in Figure 5b.

Notably, the cyclic curves exhibited excellent consistency throughout the testing process. Specifically, when the first cycle was compared with the 100th cycle, the maximum compressive strength showed only a slight decrease of 0.5 kPa, thereby underscoring the material’s remarkable elastic recovery capability.

**Figure 6:**
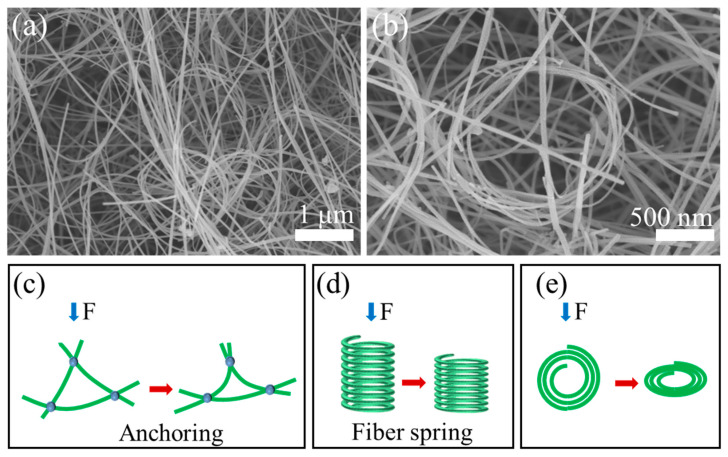
(**a**,**b**) SEM characterization of Si_3_N_4_ nanofibers; (**c**–**e**) schematic representation of the mechanism underlying enhanced compression resistance.

On the one hand, the Si_3_N_4_ nanofibers inherently possess high aspect ratios and spontaneously coil into spring-like configurations during growth on the surface of MVa, as depicted in Figure 6d,e. On the other hand, the SNP-R-SNFA exhibits two distinctive features: a unique gradient porous structure and a nanoparticle-fiber interwoven architecture. Within the low-strain regime (20–40%), the material demonstrates elastic-dominated reversible deformation, wherein energy dissipation occurs via synergistic mechanisms: (i) progressive elastic collapse of micropores and (ii) the gradual unwinding of helical spring-like nanofiber. Simultaneously, Si_3_N_4_ nanoparticles anchored at nanofiber junctions through a “pinning effect”, as demonstrated in Figure 6c, which suppress nanofiber slippage and maintains the structural coherence of the network [23,25]. These coupled mechanisms engender exceptional cyclic repeatability, as evidenced by about 2% variation in loading curve hysteresis over 100 cycles. When the applied strain reaches 60–80%, the material transitions into an elastoplastic mixed deformation regime [26]. Within this regime, three interdependent mechanisms govern mechanical behavior: (i) load transfer to dense networks: the P-side nanofiber network becomes the primary load-bearing component, resisting further compression through nanofibers bending and junctions sliding. (ii) Plastic damage initiation: partial spring-like nanofibers exceed their elastic limits, undergoing irreversible plastic relaxation. Concurrently, nanoparticle-nanofiber interfaces experience stress-induced micro-slip or partial debonding, reducing interfacial binding energy. (iii) Energy dissipation transition: cumulative plastic damage and residual pore closure jointly drive a gradual downward shift in loading curves during cycling, signifying a dominant plastic dissipation mode replacing elastic energy storage [27,28]. Critically, the submicron porous architecture (P-side) prevents catastrophic failure through stress redistribution at nanofiber junctions, while the gradient porosity (P→H side) mitigates localized stress concentration. This unique structure is the fundamental reason why the mechanical properties of SNP-R-SNF outperform those of single-structured nanofiber aerogels reported in the literature [23,24,29,30].

Particularly noteworthy is the significant enhancement in structural stability achieved through the reinforcement effect of nanoparticles. These particles fill the junctions of the nanofiber network in a pearl-necklace-like interwoven pattern, providing dual benefits: they suppress nanofiber slippage through mechanical interlocking effects and share the applied load through strong covalent bonds at the interface, thus maintaining elastic recovery over multiple cycles. Additionally, the gradient distribution of pores facilitates stepwise transfer of compressive stress along the thickness direction, preventing plastic deformation caused by localized stress concentrations. This synergistic structural design, characterized by an “elastic fiber network-rigid particle node-self-adaptive pore” configuration, enables the material to achieve an optimal balance of high strength, super elasticity, and fatigue resistance across a wide range of strain levels.

### 2.3. Dielectric Properties

**Figure 7:**
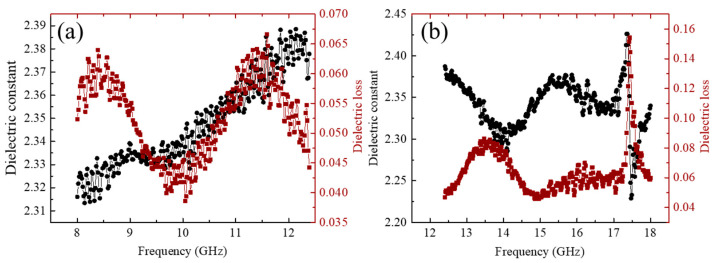
Frequency-dependent dielectric behavior of SNP-R-SNFA within the (**a**) 8.2–12.4 GHz and (**b**) 12.4–18 GHz spectral regions.

The relative complex permittivity (ε = ε′ − jε″) represents one of the crucial parameters for evaluating the dielectric properties of electromagnetic wave transmission materials. The real part (ε′) determines the polarization response and phase delay of wave—transparent materials to electromagnetic waves, while the imaginary part (ε″) characterizes the attenuation of electromagnetic waves caused by energy loss [31,32]. Therefore, to achieve excellent electromagnetic wave transparent performance, the material must possess both low values of real and imaginary parts to optimize impedance matching, reduce reflection loss and internal loss, ensuring maximum electromagnetic wave transmittance. Figure 7 illustrates the curves of the permittivity and loss tangent (tan δ = ε″/ε′) of SNP-R-SNFA varying with frequency (8.2 GHz–18 GHz). To clearly observe the changes in permittivity and loss tangent at each frequency point, a small-range vertical coordinate was adopted in plotting. It can be observed that within the frequency range of 8.2 GHz–12.4 GHz, the permittivity of SNP-R-SNFA is very small and basically remains stable, ranging between 2.31 and 2.39, while the loss tangent basically stays within 0.04–0.06. Except for an occasional significant fluctuation at 17.3 GHz, the permittivity and loss tangent show minimal variation in the 12.4 GHz–18 GHz range. However, the loss tangent increases compared to the 8.2 GHz–12.4 GHz range, predominantly remaining between 0.06 and 0.08.

Within the frequency range of 8.2 GHz–12.4 GHz, the stable low permittivity (ε’ = 2.31–2.39) benefits from the porous framework constructed by the three-dimensional nanofiber network, which effectively reduces the polarization density [33,34]. Moreover, the intrinsic broadband gap characteristic (~5 eV) of the high-purity Si_3_N_4_ crystal phase in the SNP-R-SNFA suppresses the conduction loss dominated by electron migration, keeping the loss tangent at a relatively low level of 0.04–0.06. When the frequency rises to 12.4 GHz–18 GHz, the increase in loss tangent (0.06–0.08) is mainly related to the enhancement of interface polarization: the thin silica layer (~2 nm) formed on the surface of nanofibers interacts with the bulk material, and the interface charges undergo relaxation dissipation under higher frequency electric fields [35,36]. Meanwhile, the local electric field distortion at the contact points between nanofibers intensifies the friction loss during dipole orientation [37,38]. Notably, the excellent stability of the dielectric real part throughout the entire frequency band indicates that no significant polarization mode transition occurs in the material. This is due to the whisker-like structure of nanofibers (diameter ~50 nm) effectively suppressing the abnormal polarization response caused by size effects [39,40]. However, the dielectric properties of SNP-R-SNFA are slightly inferior to those of the pure Si_3_N_4_ nanofiber aerogels reported in the literature, as their extremely low density makes the dielectric properties close to those of air. By contrast, the density of SNP-R-SNFA is 0.033 g/cm^3^, which is higher than that of most pure Si_3_N_4_ nanofiber aerogels reported in the literature [23,24]. This is one of the reasons for its relatively larger dielectric constant and loss values. On the other hand, the contact points between nanoparticles and nanofibers enhance dipole orientation loss due to local electric field distortion, leading to an increase in loss tangent, which in turn increases the absorption and scattering of electromagnetic waves. Second, the size and distribution of pores in the porous structure are crucial for electromagnetic wave transparent performance [9]. Since there are differences in the microstructures of the P-side and H-side of Si_3_N_4_ nanofiber films in each layer, the structural continuity is affected, causing multiple reflections and internal loss, thus reducing the wave-transparent efficiency. Fortunately, the side effects caused by nanoparticle aerogels are relatively small. The SNP-R-SNFA still exhibits “double-low” dielectric characteristics (low ε’ & low tan δ) over a wide frequency band. More significantly, the nanoparticles play an important role in enhancing mechanical and thermal insulation properties.

### 2.4. Thermal Insulation Characterizations

Excellent high-temperature insulation performance is crucial for radomes applied to high-Mach-number aircraft. It can prevent the high temperature generated by the friction between the radome surface and the atmosphere from being conducted to the inside of the radome, thus ensuring that the antenna works in a normal temperature environment [41,42]. Elastic Si_3_N_4_ nanofiber aerogels have excellent dielectric properties and can be used as the lining material of radomes, significantly improving their thermal insulation ability without affecting the electromagnetic wave-transparent performance of the radomes.

**Figure 8:**
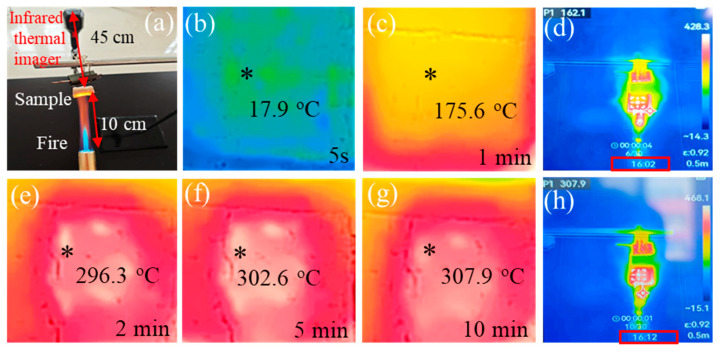
(**a**) Schematic diagram of the experimental configuration; (**b**,**c**), and (**e**–**g**) time-resolved infrared thermograms of the rear surface recorded at 5 s, 1 min, 2 min, 5 min and 10 min intervals; (**d**,**h**) original images from the infrared thermal imager window at 10-min intervals. (The asterisk (*) represents the temperature acquisition area).

Currently, researchers in this field commonly use butane flames to burn the surface of nanofiber aerogels to simulate high-temperature service environments [43,44,45]. Meanwhile, high-temperature infrared thermal imaging camera is concurrently utilized to record the temperature change and distribution status of the back-heat surface of the materials in real time. Although this method is a nonstandard characterization means, it can reasonably reflect the thermal insulation performance of materials in a high-temperature environment. The test platform built in this study is shown in Figure 8a. The thickness of the tested sample is 1 cm, and the distance between the butane torch and the sample is 10 cm to ensure that the sample surface can be fully burned by the outer flame. The distance between the high-temperature infrared thermal imaging camera and the tested sample is 45 cm. The commercial butane torch could heat the surface of the nanofiber aerogel to about 1200 °C. As illustrated in Figure 8c, during the initial 1-min combustion period, the maximum temperature recorded on the backside surface of the sample escalated to 175.6 °C. Subsequently, upon extending the combustion duration to 2 min, a rapid thermal surge was observed, with the peak backside temperature dramatically rising to 296.3 °C, as documented in Figure 8e. Following this thermal progression, after maintaining the combustion conditions for an additional 3 min (cumulatively 5 min), the maximum backside temperature plateaued at 302.6 °C, as demonstrated in Figure 8f. Notably, as evidenced by Figure 8g, even under prolonged thermal exposure spanning 10 continuous minutes, the backside temperature exhibited minimal incremental growth, registering only a 5.3 °C elevation (from 302.6 °C to 307.9 °C), thereby demonstrating exceptional thermal stabilization characteristics under sustained combustion stress. Moreover, to verify the reliability of the test data, two original infrared thermal images captured ten minutes apart were provided, as shown in Figure 8d,h. The highest temperature of the backside was about 893 °C lower than that of the heated surface (the nominal value of butane flamethrower), highlighting its excellent thermal insulation performance.

In practice, the pore diameters of pure Si_3_N_4_ nanofiber aerogels reported in the current literature are generally several micrometers or even tens of micrometers [23,24,46]. While their thermal insulation performance remains critically dependent on ultralow densities, excessive density reduction precipitates mechanical degradation, limiting practical utility. As revealed by the microstructural analysis in Section 2.1, the superior thermal insulation performance of the SNP-R-SNFA originates from its gradient pore architecture and nanoparticle-nanofiber synergistic reinforcement. The pearl-necklace-like composite structure (nanofibers and nanoparticles intertwined) on the H-side synergistically suppresses heat transfer via dual mechanisms: (1) nanoparticle-aerogel subdivision of micron-scale pores into nano/submicron domains, prolonging gas molecular mean free paths to inhibit convection; (2) heterogeneous Si_3_N_4_ nanofiber/Si_3_N_4_ nanoparticle interfaces and surface defects (such as dangling bonds) that enhance phonon scattering, reducing solid-phase thermal conductivity [47,48]. The high-density nanofiber network on the P-side further restricts gas motion through the Knudsen effect while reflecting infrared radiation via its compact architecture [49,50]. More importantly, the gradient density distribution from the P-side to the H-side (nanofiber density decreases and porosity increases) establishes a continuously graded refractive index interface, effectively weakening the Fresnel reflection loss of thermal radiation and achieving wide-spectrum radiation heat shielding [51,52]. This multi-scale structural design ultimately makes the SNP-R-SNFA exhibit excellent thermal insulation performance in a wide temperature range by hierarchically regulating the three heat transfer paths of gas, solid, and radiation. It achieves superior thermal insulation performance at a higher density (0.033 g/cm^3^), significantly enhancing its practical applicability compared to those nanofiber aerogels that rely excessively on ultralow density for thermal insulation.

## 3. Conclusions

This study pioneered a gas-phase self-assembly strategy to fabricate SNP-R-SNFA with gradient pore architectures. The MTa and MVa organosilane aerogels were initially synthesized as parent matrices for generating Si_3_N_4_ nanofiber films, and the effects of the microstructure and composition of organosilane aerogels on the microstructure of Si_3_N_4_ nanofiber films were investigated. By leveraging MVa as a reactive template, SNP-R-SNF featuring nanoparticle-nanofiber interpenetration and porosity gradients was prepared. The microstructure formation mechanism of SNP-R-SNF was analyzed in detail. Layer assembly and hot-pressing composite processing were employed to prepare the SNP-R-SNFA, which showed low density (0.033 g/cm^3^), exceptional compression resilience, superior dielectric stability (ε′ = 2.31–2.39, tan δ < 0.08 across 8–18 GHz), and outstanding thermal insulation performance. This study not only provides material solutions for integrated electromagnetic wave-transparent/thermal insulation particularly for high-Mach-number aerospace applications, but more importantly establishes an innovative paradigm for enhancing the mechanical robustness of nanofiber-based aerogels.

## 4. Materials and Methods

### 4.1. Materials

Methyltrimethoxysilane (MTMS, AR), triethoxyvinylsilane (VTES, AR), N, N-dimethylformamide (DMF, AR), and aqueous ammonia (25–28%) were procured from Shanghai Aladdin biochemical technology Co., Ltd. (Shanghai, China). Acetic acid (CH_3_COOH, AR) and n-Hexane (AR) were sourced from Shanghai Maclin biochemical technology Co., Ltd. (Shanghai, China). Deionized water and absolute ethanol were supplied by Shanxi Hamp technology Co., Ltd. (Taiyuan, China). All chemicals and solvents were utilized directly without additional purification steps.

### 4.2. Preparation of Organosilane Aerogels

Two organosilane aerogels were synthesized: a pure methyltrimethoxysilane aerogel (MTa) and an MTMS/vinyltriethoxysilane (VTES) composite aerogel (denoted as MVa, with a molar ratio of MTMS:VTES = 2:1. The precursor molar ratio was (MTMS + VTES):ethanol:H_2_O:CH_3_COOH:DMF = 1:3:4:0.75:0.2. The synthesis process commenced with sequential addition of deionized water, anhydrous ethanol, DMF, and VTES into a glass beaker, followed by 30 min of magnetic stirring at 200 rpm. MTMS was subsequently introduced, and glacial acetic acid was added dropwise under continuous agitation. The mixture underwent hydrolysis in a 30 °C water bath for 24 h to form a homogeneous sol. The gelation phase was initiated by dropwise addition of aqueous ammonia under intensified stirring (400 rpm) until the sol pH reached 9, after which the stirring rate was reduced to 200 rpm for 10 min to stabilize the colloid before transferrin into molds. Following 12 h of gel maturation, the wet gels were demolded and aged in anhydrous ethanol at 60 °C for 48 h, with ethanol replenishment every 12 h. Subsequent solvent exchange with n-hexane was performed through four cycles (6 h per cycle) to eliminate residual polar components. Finally, the gels were vacuum-dried at 60 °C for 24 h, yielding monolithic organosilane aerogels.

### 4.3. Preparation of the SNP-R-SNFA

The MTa and MVa were placed at the bottom of a graphite crucible engineered with multiple lateral apertures to optimize N_2_ permeation, facilitating efficient carbothermal reduction nitridation within a controlled atmosphere furnace. A thermal regimen of 5 °C/min heating/cooling rates was implemented, maintaining a 3 h isothermal hold at 1450 °C under 0.1 MPa N_2_ pressure to ensure complete reaction. Then, the organosilane aerogels were transformed into Si_3_N_4_ aerogels, accompanied by the spontaneous formation of a layer of Si_3_N_4_ nanofiber film on their surfaces. After that, the Si_3_N_4_ nanofiber film was carefully peeled off from the surface of the Si_3_N_4_ aerogel by a simple mechanical exfoliation method. The stripped Si_3_N_4_ films were superimposed layer by layer in the direction of the inner side (the side in contact with the Si_3_N_4_ aerogel), and the hot-pressing composite was finally performed to prepare SNP-R-SNFA.

### 4.4. Characterization

The bulk density of SNP-R-SNFA was determined gravimetrically through mass-to-volume ratio calculations based on geometric dimensions. Phase composition analysis was conducted via X-ray diffraction (XRD, D/Max-2500, Rigaku, Tokyo, Japan) using Cu-Kα radiation (λ = 1.5406 Å) with a scan rate of 2°·min^−1^ between 10–80°. Microstructural characterization was performed using field-emission scanning electron microscopy (FE-SEM, S4800, Hitachi, Tokyo, Japan) at an acceleration voltage of 5 kV, coupled with energy-dispersive X-ray spectroscopy (EDS) for elemental mapping. Quasi-static compressive testing was executed on a universal testing system (WDW-50, Jinan Kai Rui, Jinan, China) equipped with a 5 kN load cell. Stress–strain profiles were acquired under displacement control at 10 mm·min^−1^ crosshead velocity, with nominal strain levels incrementally increased to 20%, 40%, 60%, and 80% of original height. Cyclic fatigue resistance was evaluated through 100 consecutive compression cycles at 40% constant strain amplitude, employing an elevated strain rate of 100 mm·min^−1^ to simulate dynamic loading conditions. Frequency-dependent dielectric permittivity and loss tangent were measured at ambient conditions using a vector network analyzer (AV3672D, Qingdao, China) with rectangular waveguide fixtures (X-band: WR-90, 22.86 × 10.16 × 3.5 mm^3^; Ku-band: WR-62, 15.80 × 7.90 × 3.5 mm^3^) across 8.2–18 GHz. High-temperature endurance was assessed through butane torch ablation tests (flame temperature: 1200 °C) applied to the frontal surface, while transient thermal gradients were monitored using an infrared thermographic camera (H21ProS+, HIKMICRO, Hangzhou, China) with 512 × 384-pixel resolution and ±2% measurement accuracy.

## Data Availability

The original contributions presented in this study are included in the article. Further inquiries can be directed to the corresponding author.
